# Future Nursing Home Placement, Health, and Relationships by Sexual Orientation: Findings From the HRS

**DOI:** 10.1093/geroni/igaa057.2342

**Published:** 2020-12-16

**Authors:** Mekiayla Singleton, Zach Gassoumis, Susan Enguidanos

**Affiliations:** University of Southern California, Los Angeles, California, United States

## Abstract

This study compares LGB and heterosexual adults on anticipated need for future nursing home (NH) placement and factors that influence NH placement. Using data from the 2016 HRS, we found a trend toward higher anticipated NH placement among LGB adults (M=34.0, SD=29.2) as compared to heterosexual adults (M=26.9, SD=27.3; p=0.05). Compared with LGB respondents (n=137), heterosexual adults (n=3,469) were more likely to have living child/children (37% vs. 82%, p<.001). Although there was no statistical difference in marital status between LGB (50% married/partnered) and heterosexual adults (65% married/partnered; p=.06), married/partnered LGB respondents were more likely to be very/quite close to their spouse/partner (99% vs. 90%, p<0.01) compared to heterosexual respondents. There was no difference in number of health conditions between groups. This study suggests that LGB adults may have higher rates of anticipated need for NH, thus NHs must ensure their workforce is educated and prepared to support this growing population. Part of a symposium sponsored by Rainbow Research Group Interest Group.

